# Amylose starch with no detectable branching developed through DNA-free CRISPR-Cas9 mediated mutagenesis of two starch branching enzymes in potato

**DOI:** 10.1038/s41598-021-83462-z

**Published:** 2021-02-22

**Authors:** Xue Zhao, Shishanthi Jayarathna, Helle Turesson, Ann-Sofie Fält, Gustav Nestor, Matías N. González, Niklas Olsson, Mirela Beganovic, Per Hofvander, Roger Andersson, Mariette Andersson

**Affiliations:** 1 Department of Molecular Sciences, Swedish University of Agricultural Sciences, Box 7015, 750 07 Uppsala, Sweden; 2 Department of Plant Breeding, Swedish University of Agricultural Sciences, P.O. Box 101, 23053 Alnarp, Sweden; 3 Consejo Nacional de Investigaciones Científicas Y Técnicas, (C1425FQB), Buenos Aires, Argentina; 4 Laboratorio de Agrobiotecnología, IPADS (INTA - CONICET), Ruta 226, Km 73.5, B7620 Balcarce, Argentina

**Keywords:** Biotechnology, Plant sciences

## Abstract

DNA-free genome editing was used to induce mutations in one or two branching enzyme genes (*Sbe*) in tetraploid potato to develop starch with an increased amylose ratio and elongated amylopectin chains. By using ribonucleoprotein (RNP) transfection of potato protoplasts, a mutation frequency up to 72% was achieved. The large variation of mutations was grouped as follows: Group 1 lines with all alleles of *Sbe1* mutated, Group 2 lines with all alleles of *Sbe1* as well as two to three alleles of *Sbe2* mutated and Group 3 lines having all alleles of both genes mutated. Starch from lines in Group 3 was found to be essentially free of amylopectin with no detectable branching and a chain length (CL) distribution where not only the major amylopectin fraction but also the shortest amylose chains were lost. Surprisingly, the starch still formed granules in a low-ordered crystalline structure. Starch from lines of Group 2 had an increased CL with a higher proportion of intermediate-sized chains, an altered granule phenotype but a crystalline structure in the granules similar to wild-type starch. Minor changes in CL could also be detected for the Group 1 starches when studied at a higher resolution.

## Introduction

Genome editing in plants has opened up new possibilities, both in terms of research and development of crops with novel traits beneficial for human health or the environment. The method can be used to create knockouts of enzymatic functions, which in many cases has been difficult to achieve with traditional genetic engineering methods, e.g. RNAi. Protocols for genome editing through CRISPR-Cas9 have been successfully established for inducing mutations in potato^1^. In addition, using transient approaches for genome editing of potato is possible, which is a big advantage when plants free of recombinant DNA are desired. A transient approach has a clear benefit for highly heterozygous clonally propagated crops like potato, since segregation through seed generations is more or less impossible^2^.

Potato is an important, high-yielding, nutritious and starch-rich staple crop^3^. Starch is composed of glucose units linearly linked by α(1 → 4) glycosidic bonds and branched by α(1 → 6) bonds. Starch is generally deposited as highly ordered granules directed by the highly branched amylopectin molecule with the addition of the essentially linear molecule amylose to a ratio of approximately 4:1^4^. In food products, the high amylose content and the long chains of amylopectin contribute to formation of resistant starch and relate to a low glycaemic index (GI) after intake^5,6^. By increasing the average chain length of potato starch, a starch with health benefits can be developed. The increased chain length can yield resistant starch (RS) leading to a low GI and concur health benefits by promoting the growth of healthy gut flora, and lowering both the caloric intake and cholesterol levels in the blood^5^. The long-chain quality of starch also creates beneficial properties as a raw material for producing bioplastic films^7^, which in the future might replace some of the fossil-based plastics produced today.

The research and development of potatoes with novel starch qualities has been ongoing for a long time. Potatoes that synthesise solely amylopectin starch have been developed by eliminating granule-bound starch synthase (GBSS) activity through traditional mutagenesis, antisense, RNAi and most recently CRISPR-Cas9^8–11^. The development of potatoes with a high ratio of amylose starch and/or altered starch chain length distribution has been achieved by targeting two starch branching enzymes (SBEs) using traditional gene silencing technologies and recently genome editing^12–14^. A very high amylose starch content was found synthesised at the expense of total starch content and plant development^15^. Based on those results and the fact that, so far, no potato studies have resulted in pure amylose starch, it could be speculated that the presence of a fraction of amylopectin in potato starch is essential for plant development. In contrast, barley with suppressed activity of three SBEs (SBEI, SBEIIa and SBEIIb) was found to have an amylose-only starch in the endosperm^16^. In that study, the high amylose content only had a minor impact on grain yield and starch content.

In a recent study by Tuncel et al*.*^14^, CRISPR-Cas9 was used to target the two SBEs in potato. Lines with mutations in *Sbe1* or *Sbe2* alone or in combination were developed using either traditional *Agrobacterium*-mediated transformation or PEG-mediated protoplast transfection with vector DNA. In that study, lines mutated in *Sbe1* were not found affected in their starch structure, while tuber cells from *Sbe2* mutated lines displayed an increased number of granules. One line had a strong reduction in both SBEs, resulting in starch with an altered granule phenotype, longer amylopectin chains and a degree of branching that was reduced by half.

Other crops have also been targeted for developing high-amylose starch genotypes using genome editing, such as rice and sweet potato. Rice was subjected to mutagenesis in the respective *Sbe* genes through CRISPR-Cas9 and *Agrobacterium*-mediated transformation^17^. In that study, mutations in the *Sbe1* gene did not result in any major changes in the starch compared to the parental variety, while mutations in *Sbe2* led to a starch with increased amylose content from ca. 15 to 25% and an amylopectin chain-length distribution which shifted towards longer chains^17^. Similar results were obtained on sweet potato mutated in *Sbe2*, with an increase in amylose content from ca. 27 to 40%^18^.

In this study, we used a previously established CRISPR-Cas9 RNP-method to induce mutations in *Sbe1* individually and *Sbe1*–*Sbe2* simultaneously in potato. By using genome editing to induce mutations in all eight *Sbe* alleles, we were able to develop, for the first time, a unique potato starch essentially lacking branching. We further investigated the effects of this starch as well as starches with altered amylopectin structure on plant development and starch granular structure and phenotype.

## Results

### *Sbe1* and *Sbe2* targeted mutagenesis and genotyping of regenerated potato lines

*Sbe1* was targeted alone or in combination with *Sbe2* in the potato variety Desiree (Supplementary Fig. S1). Dual sgRNAs named BE1T3, BE1T4 (*Sbe1*) and BE2T3 and BE2T4 (*Sbe2*) (Supplementary Fig. S1c) were preassembled with Cas9 and transfected to potato protoplasts as ribonucleprotein complexes (RNPs). In the single gene target experiment, 221 regenerated shoots were analysed using high-resolution fragment analysis (HRFA), while in the stacking gene target experiment, 68 regenerated lines were analysed. The experiments had a mutation frequency of 52% and 72% respectively, calculated based on the number of lines where at least one allele was mutated (Supplementary Table S2). Thirteen lines were selected for further study and genotyped using Sanger sequencing to confirm the insertions/deletions (indels) size and to investigate the genomic structure of the mutations (Table 1). Based on the number and combination of alleles mutated, the lines were divided into three groups: five lines had mutations in all four alleles of *Sbe1*, 82007, 82050, 82079, 104011 and 104032 (Group 1), six lines had four-allele mutations in *Sbe1* combined with two to three alleles mutated in *Sbe2,* 104001, 104005, 104006, 104016, 104018 and 104034 (Group 2) and two lines, 104010 and 104023, had all eight alleles mutated (Group 3). Both lines in Group 3 contained in-frame indels in the *Sbe1* and/or *Sbe2* mutated alleles (Table 1, Supplementary Table S3).

**Table 1:** Size of indels in respective line analysed with Sanger sequencing and HRFA (in brackets), where “0” indicates the wild type allele fragment size, “–” represents a deletion and “ + ” represents an insertion.

Group	Line	Size of indels confirmed by Sanger sequencing (results from HRFA in brackets)	
		Sbe1	Sbe2
Group 1	82007	-38^a^/-3^¥,a^/ + 70^a^ (-38/-3/ + 70)	0^¤^ (0)
	82050	-22^a^/-1^a^/ + 38^a^/ + 165^¥,a,e^ (-22/ -1/ + 38/ + 166)	0^¤^ (0)
	82079	-1^a^/ + 47^a,e^ (-1/ + 47)	0^¤^ (0)
	104011	-94^a,b^/-4^a^ (-95/-4)	0^¤^ (0)
	104032	-55^a,b^/ + 26^a,b^ (-56/ + 26)	0^¤^ (0)
Group 2	104001	-94^a,b^ / + 3^¥,a,b^ (-95/ + 3)	-129^¥,c,d^/-3^¥,c,d^/-1^c^/0^¤^ (-130/-3/-1/0)
	104005	-93^¥,a,b^/-92^,a,b^ (-94/-93)	-10^d^/-4^c,d^/0^¤^ (-10/-4/0)
	104006	-92^a,b^/-5^a,b^/-1^a^ (-92/-5/-1)	-11^d^/-1 ^d^/0^¤^ (-11/-1/0)
	104016	-93^¥,a,b^/-4^a,b^ (-94/-4)	-2^c^/0^¤^/ + 6^¥,d^ (-2/0/ + 6)
	104018	-93^¥,a,b^/-23^a,b^/-17^a^/ + 153^¥,a,b^ (-94/-23/-17/ + 153)	-1^c^/0^¤^/ + 104^c,d^ (-1/0/ + 105)
	104034	-94^a,b^ /-93^¥,a,b^ /-5^a,b^ / + 60^¥,a,b^ (-95/-94/-5/ + 60)	-8^c,d^/-6^¥,c,d^/0^¤^ (-8/-6/0)
Group 3	104010	-5^a,b^/ + 92^a,b^/ + 123^¥,a,b^ (-5/ + 93/ + 122)	-2^d^/-1^c^/ + 48^¥,c,d^/ + 194^c,d^ (-2/-1/ + 48/ + 195)
	104023	-92^a,b^/-3^¥,a,b^/ + 13^a,b^ / + 251^a,b^ (-92/-3/ + 13/ + 252)	-127^c,d^/-9^¥,c,d^/-5^c,d^ (-128/-9/-5)

Less than four different indels indicates that at least two alleles share the same genetic context. Lack of “0” means no wild type allele remaining in the line. Group 1 represents lines mutated in all four alleles of *Sbe1*; Group 2 represents lines mutated in all four alleles of *Sbe1* and two to three alleles in *Sbe2*; Group 3 represents lines mutated in all alleles of both *Sbe1* and *Sbe2*. ^¤^ Wild type alleles. ^¥^ In-frame indels. ^a^ Indels at BE1T3 target site, ^b^ Indels at BE1T4 target site, ^c^ Indels at BE2T3 target site and ^d^ Indels at BE2T4 target site. ^e^ Insert origination from vector used for in vitro transcription.

Sanger sequencing results were in line with the results from the HRFA except for large indels, where a 1 bp difference could occasionally be noted (Table 1). The majority of the lines had at least one allele with a large deletion in *Sbe1* due to dual sgRNA mediated cuts, but only one of them, 104005, had a large deletion in all four alleles. The frequency of large deletions due to dual cuts was considerably lower in *Sbe2*. In alleles with mutations not corresponding to a predicted large deletion, indels could be found at both target sites of the sgRNA pair (Table 1). No indels could be observed in one of the alleles of the BE2T4 target region, which had a 1 bp mismatch directly adjacent the protospacer adjacent motif (PAM) site (Supplementary Fig. S1c).

### Yield, dry matter, tuber phenotype and sprouting of greenhouse-grown tubers

The thirteen selected lines were grown in a greenhouse until senescence. As comparators during the greenhouse trial and subsequent analyses, the parental variety Desiree and a high-amylose RNAi line T-2012 were included.

Tubers from the Group 3 lines were clearly reduced in size and had a significant total tuber yield drag per plant of 60–80% compared to the parental variety (Supplementary Fig. S2b,c and S3d,e). Further conclusions concerning the number of tubers, total tuber yield per plant and average tuber weight cannot be drawn since the results fluctuated considerably between the lines and biological replicates (Supplementary Fig. S2a–c).

Tuber phenotype was unaffected in Group 1 lines compared to the parental variety (Supplementary Fig. S3a,g), while tubers in the Group 2 lines had some additional buddings detected (Supplementary Fig. S3b,c). The most dramatic phenotypical differences compared to parental variety were found in the Group 3 lines, where tubers were small and elongated with numerous additional buddings from the main tuber (Supplementary Fig. S3d,e). Tubers from the Group 3 lines had a significant decrease in dry matter content (Supplementary Fig. S2d), a consequence of smaller and decreased number of starch granules in the tuber cells (Fig. 6f) compared to the comparators and lines from Group 1 and 2 (Fig. 6b,d,h,j). The dry matter content of the tubers from Group 1 and 2 was not significantly different from the parental variety (Supplementary Fig. S2d).

Sprouting of harvested tubers was studied after five months in cold storage. Tubers from all lines already had sprouts initiated at the end of the cold storage period, which continued to develop further at room temperature with no major differences among the lines (Supplementary Fig. S4).

### Amylose content and chain-length distribution of tuber starch

**Figure 1:**
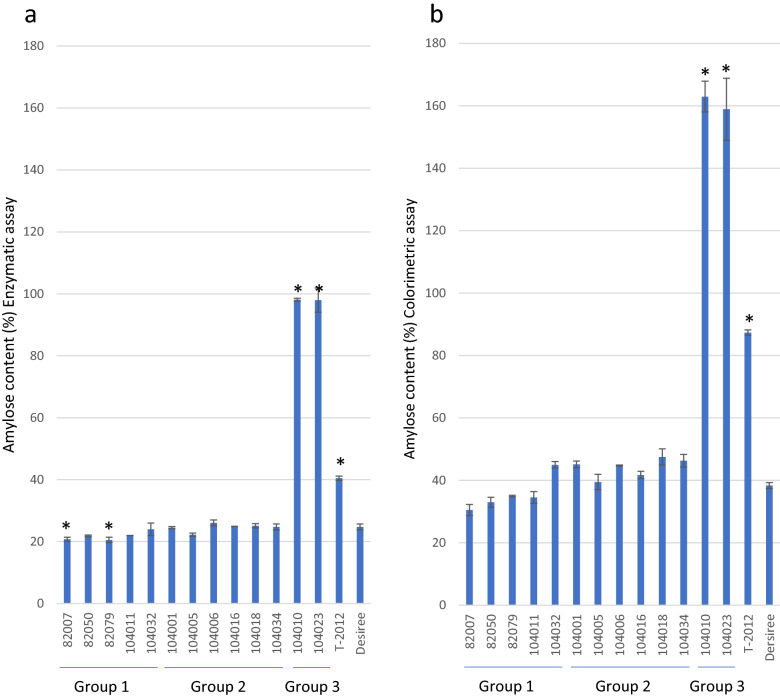
Amylose content of extracted starches measured using a. enzymatic assay and b. colorimetric assay. The results are a mean of two technical replicates, error bar represents standard deviation (s.d.). Values that differ from the parental variety Desiree by Dunnett’s test (P < 0.05) are marked with *.

Starch was isolated from tubers harvested from the thirteen mutated lines and their comparators. Starch quality and structure was studied using several methods. Based on an enzymatic method, the amylose content was found to be 98% in both lines in Group 3 (Fig. 1). No significant increase in amylose could be found for lines in Groups 1 and 2 compared to the parental variety (25% amylose), while the RNAi line T-2012 showed an amylose content of 40%. The amylose content was also measured using a colorimetric method, which was found to be more influenced by variations in the chain length distribution of the amylopectin molecule but overestimating the amylose ratios (Fig. 1b). This method yielded an amylose content of 159–168% in the lines of Group 3. An intermediate amylose content between 40 and 48% was measured in the Group 2 lines. The majority of the lines in Group 1 was found to be ranging from 31 to 35%, which was somewhat lower than for the parental variety having an amylose content of 38%. An exception was line 104032 in Group 1, which was determined to have a 45% amylose content. The amylose content of the RNAi line was measured to be 87%, which is close to the originally published results of 89% for T-2012 using the same colorimetric method.

**Figure 2:**
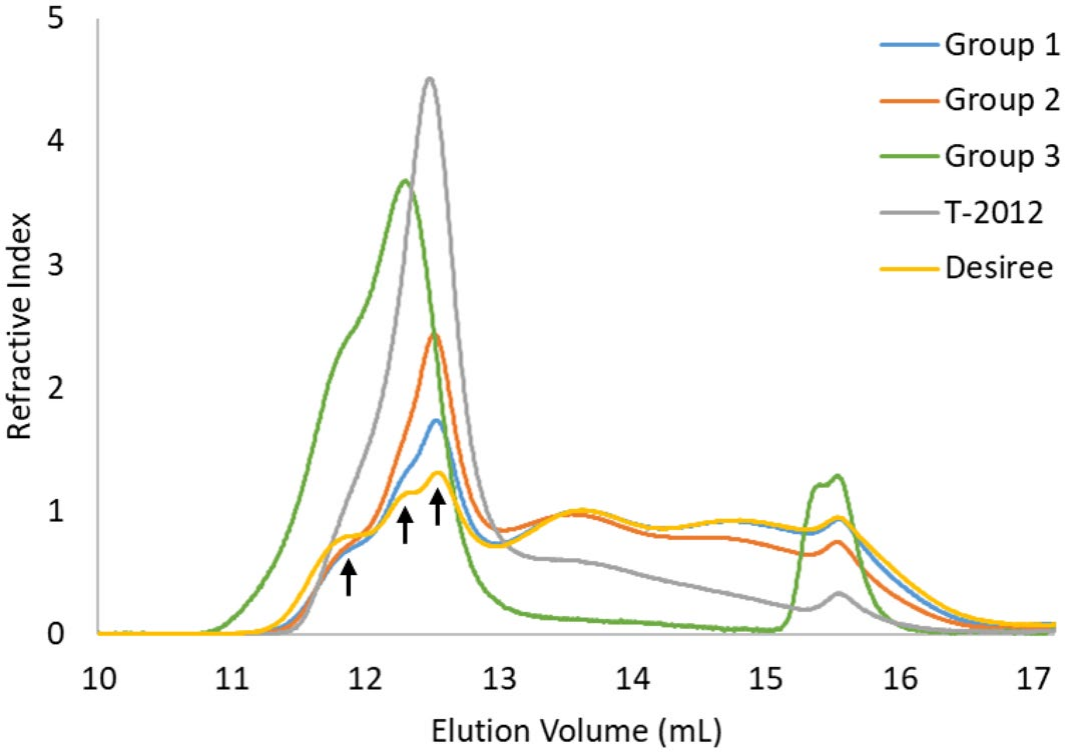
Chain-length distribution of debranched starches from the potato lines after normalisation for the peak area, analysed with HPSEC. The averages of the potato lines from Groups 1, 2 and 3 are shown. The parental variety Desiree and the high-amylose line T-2012 were included for comparison. The arrows from left to right point out the three populations of amylose chains, i.e. long, intermediate, and short chain amylose fraction, respectively. Software used is ASTRA software version 4.70.07 (wyatt.com/products/software/astra.html, Wyatt Technology Corp., Santa Barbara, CA).

High-performance size exclusion chromatography (HPSEC) and high-performance anion exchange chromatography (HPAEC) were used to investigate the chain-length distribution of debranched starches. In the HPSEC analysis, the debranched starch samples of Group 1 lines showed a similar chain-length distribution pattern as the parental variety (Fig. 2) but with slightly different amounts for different fractions of amylose. An exception was line 104032, whose chain-length distribution pattern was close to the Group 2 starches (Supplementary Fig. S5). Based on calculations using MALLS, it could be seen that the molecular weight at around 13 mL is in the order of 100,000 g/mol (Supplementary Fig. S6). The molecular weight of amylose generally ranges from about 80,000 to about 1,000,000 g/mol^19^, which indicates that the fraction eluted before an elution volume of 13 mL is the major amylose fraction and the fraction eluted after an elution volume of 13 mL is the major amylopectin fraction. Three peaks were eluted between 11 and 13 mL in the starch from the parental variety, which may correspond to three different chain lengths of amylose and are referred as long, intermediate and short-chain amylose fractions hereafter.

Substantial changes to the chain-length distribution pattern, both in the amylose and amylopectin fractions, could be observed for the lines of Group 2 and Group 3 compared to the parental variety (Fig. 2). The peak, which may correspond to the intermediate amylose, is smaller for the lines in Group 2. Alterations in the chain-length distribution were most prominent in Group 3 where the amylopectin fraction between the elution volume of 13 mL and 15 mL was absent and compensated by clearly elevated fractions of amylose. However, compared to the parental variety, these lines lacked the population that may correspond to the short-chain amylose fraction. Moreover, an extra peak appeared at an elution volume of 15.4 mL, which in theory represents a short-chain amylopectin fraction (DP > 6). However, it was not possible to observe any peak for maltodextrins (DP > 6) from the HPAEC analysis for the starches from Group 3. This limits the possibility that the extra peak is associated with a short-chain amylopectin fraction. The T-2012 RNAi-line had a chain-length distribution pattern that differs from all other lines with an altered amylopectin chain-length distribution and an elevated fraction of amylose or amylose-like long glucan chains (Fig. 2).

**Figure 3:**
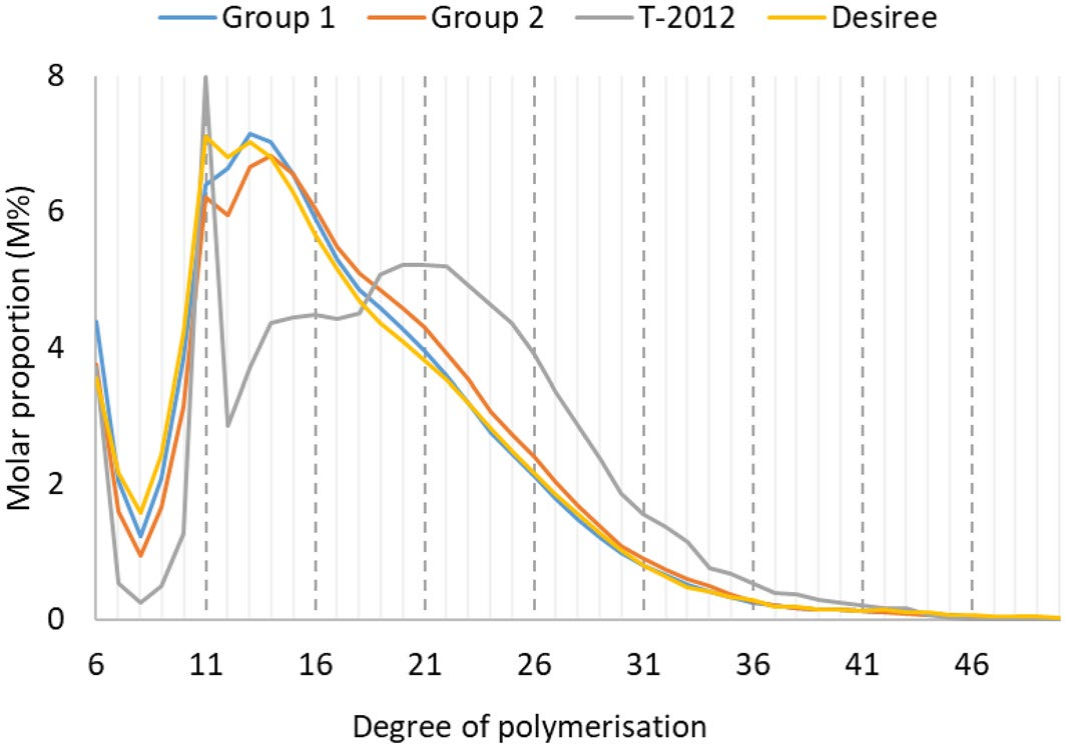
Chain-length distribution of debranched starches on a relative molar basis (M%) with degree of polymerization (DP) 6–50, based on HPAEC analysis with averages of potato lines from Groups 1 and 2. No peak was detected for the starches from Group 3. The parental variety Desiree and the high-amylose line T-2012 were included for comparison.

The molar proportion distribution of different chain lengths of starch from the potato lines, analysed by HPAEC, is given in Fig. 3. Starch from Desiree showed a pattern where there is a predominant broad peak of chains spanning the DP ~ 9–33 range, with a shoulder at DP11 and a slight increase of chains from DP18 (Fig. 3). Starch from T-2012, however had a very different pattern of the chain length distribution, where there was a large decrease in short chains of DP7–10, a high and sharp peak of chain at DP11, and less chains of DP12–18 and more chains of DP19–42 than the other samples (Fig. 3). The proportion of chain lengths of DP ≥ 43 was very low for all the potato lines. Starch from Group 1 lines was very similar to the parental variety in the chain length distribution of long chains of DP > 33, with increased abundance of moderately sized chains of DP12–21 and reduced number of short chains of DP7–11 and intermediate-sized chains of DP22–33 (Fig. 3, Supplementary Fig S7a). The biggest effect was observed with an increase in the proportion of chains at DP6 (Supplementary Fig. S7a). In the starch from lines in Group 2, there was a decline in the proportion of short chains of DP ≤ 13, and an increase in those of the intermediated-sized chains of DP14–33 and, effects on chains of DP > 33 were not pronounced compared to the parental variety (Fig. 3, Supplementary Fig. S7b). There was no peak detected for debranched amylopectin chains from the HPAEC analysis for the starches from Group 3. Amylose is essentially long linear molecule which was beyond the separation range of HPAEC, and the chain-length distribution from the HPAEC analysis did not account the chains originating from the amylose fraction (Fig. 3).

### Degree of branching of Group 3 starches

**Figure 4:**
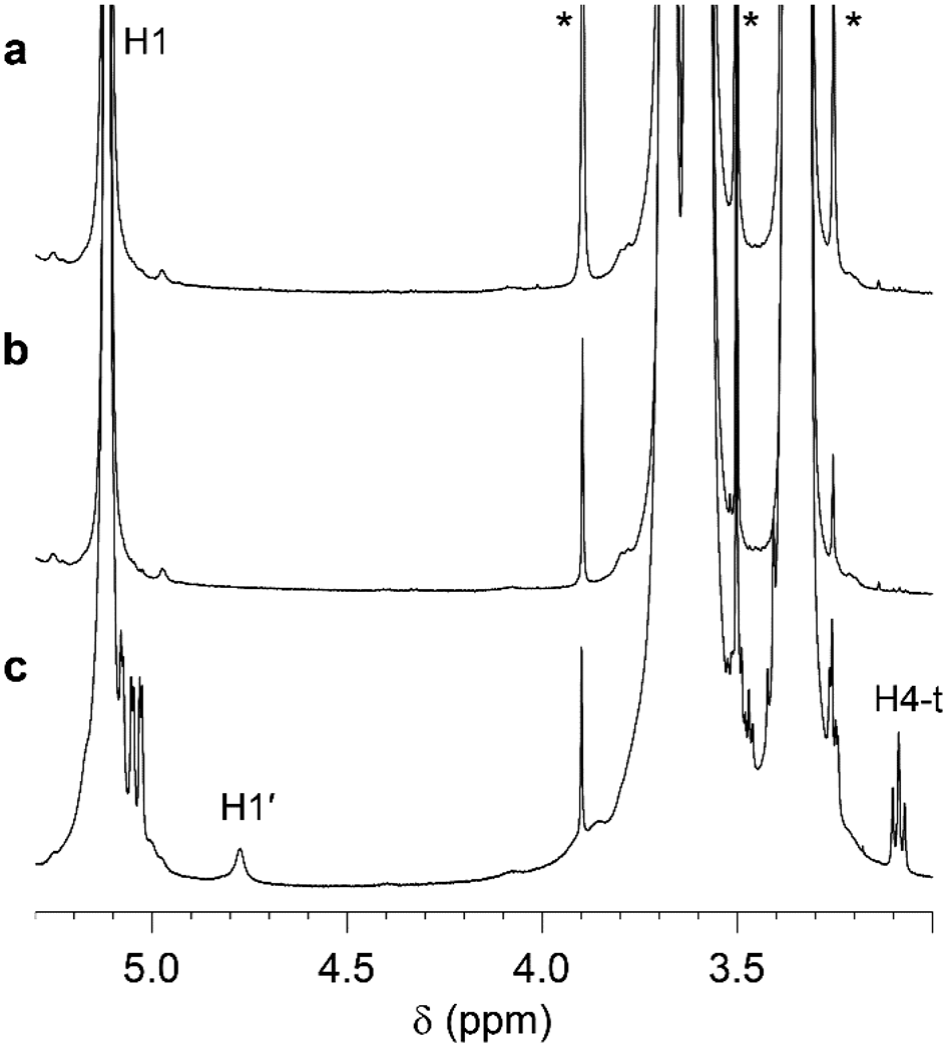
^1^H NMR spectra of starch from the Group 3 lines (**a**) 104010 and (**b**) 104023, and from (**c**) the parental variety Desiree. Glucose H1 at the α(1 → 4)-linkage (H1) and at the α(1 → 6)-linkage (H1′) are assigned as well as H4 of non-reducing end terminal residues (H4-t). Traces of EDTA and Tris are highlighted with asterisks.

The degree of branching (DB) was analysed using NMR spectroscopy on starch from lines in Group 3 and the parental variety Desiree. At the branching point, the anomeric proton at the α(1 → 6)-linkage has a different chemical shift compared to other anomeric protons and can thus be used for quantification of the DB. Neither starch from line 104010 nor 104023 showed any detectable branching (< 0.1%), whereas the starch from Desiree showed a DB of 3.0% (Fig. 4). Similarly, the amount of terminal residues was markedly different, with the non-reducing end H4 yielding a clear signal from the Desiree starch but a very weak signal from the Group 3 starches (Fig. 4).

### Crystalline type and molecular order of the starch granules

**Figure 5:**
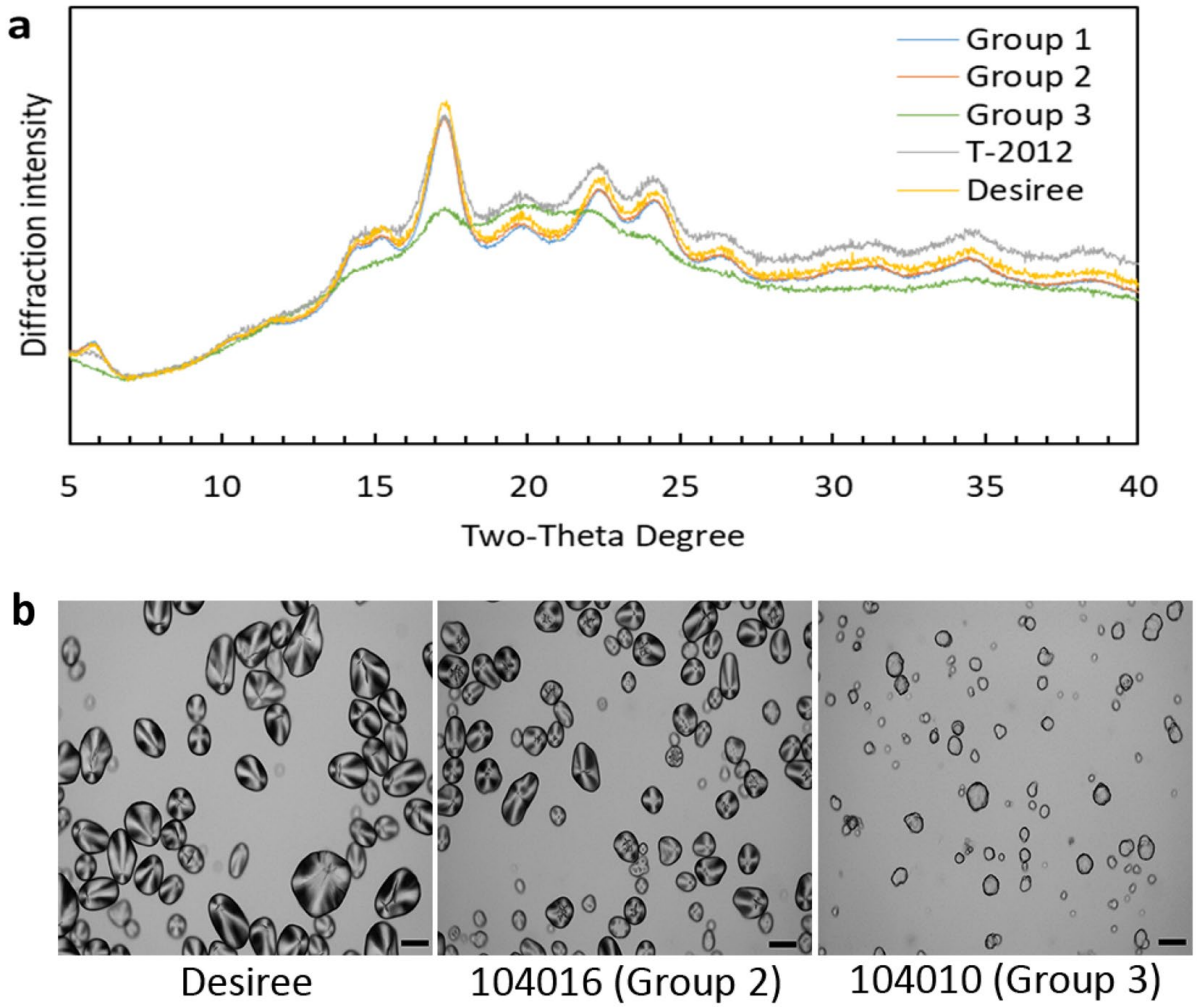
X-ray diffraction patterns and polarised light microscopy of potato lines from Groups 1, 2, and 3. (**a**) Average diffraction intensity for each group. The parental variety Desiree and the high-amylose line T-2012 were included for comparison. (**b**) Images of selected potato starches using polarised light microscopy. Scale bar = 40 µm.

As displayed in Fig. 5, most of the potato lines showed the B-type X-ray diffraction pattern^20^ with diffraction peaks at 15° (broad), 17° (strong) and a doublet at 22–24° 2θ, which is the common pattern for the tuber starches. However, the X-ray diffraction pattern and the diffraction intensities of the Group 3 lines differed from the other lines. A decrease in the diffraction intensity for most of the peaks was observed in the Group 3 lines when compared to the other lines. However, it was still possible to observe all the major diffraction peaks that correspond to B type starches (Fig. 5a). Moreover, the starch from Group 3 did not show Maltese crosses in the starch granules under polarised light, as was found in starch from the parental variety Desiree or 104016 (Fig. 5b).

### Starch granule phenotype

**Figure 6:**
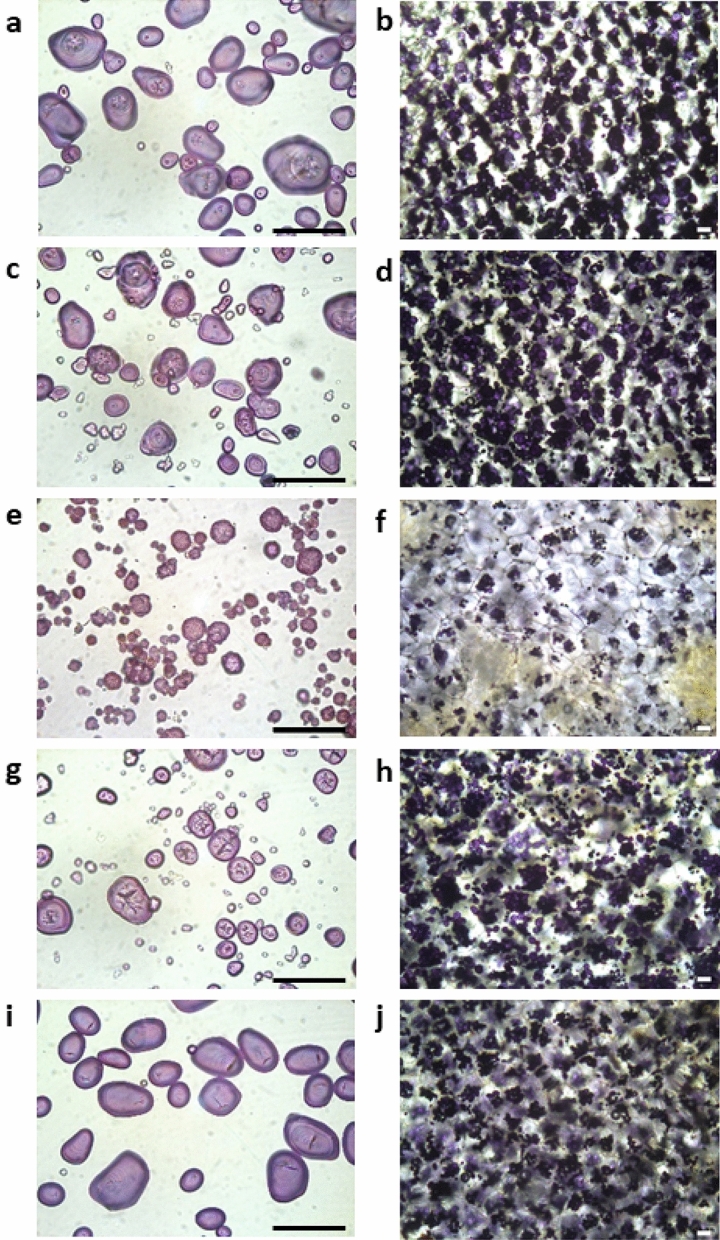
Starch granules (left images) and thin sliced tuber tissue (right images) stained with iodine and visualised under light microscope. (**a**,**b**) 82079 (Group 1) (**c**,**d**) 104018 (Group 2) (**e**,**f**) 104010 (Group 3). (**g**,**h**) RNAi line T-2012. (**i**,**j)** parental variety Desiree. Scale bar = 100 µm.

Starch stained with iodine and studied with light microscopy revealed that the granular phenotype was affected in all lines but at different levels. Starch from lines in Group 1 were most similar to starch granules from the parental variety, oval in shape but with a minor truncation in the core (Fig. 6a). The truncated core became even more pronounced in starch from the lines in Group 2 and had more irregularly shaped granules (Fig. 6c), where several of the granules had a similar phenotype as the RNAi line (Fig. 6g). Granules from the lines in Group 3 were most affected as compared to the parental line and found to have a spherical multi-lobed phenotype (Fig. 6e,i).

### Greenhouse study of green biomass and transitory starch

**Figure 7:**
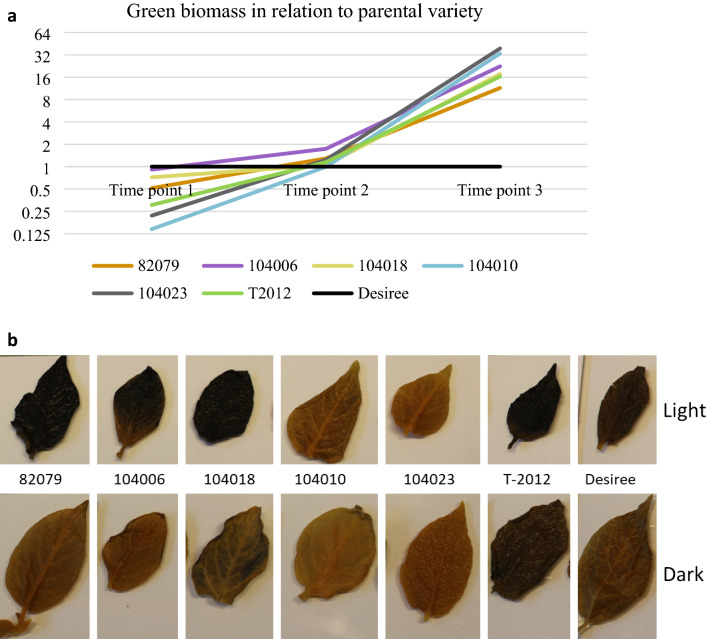
Transitory starch and green biomass of plants grown in greenhouse. Lines analysed are; 82079 (Group 1), 104006 (Group 2), 104018 (Group 2), 104010 (Group 3), 104023 (Group 3), T-2012 and parental variety Desiree. (**a**) Green biomass measured with low-cost RGB imaging phenotyping lab using digiCamControl (digiCamControl v2,1,2, http://www.digicamcontrol.com) and Easy Leaf Area. All lines are normalised to the parental variety Desiree, which is set to 1. The results are a mean of three biological replicates. Note that the vertical axis has a logarithmic scale. (**b**) Leaf tissue of top shoots harvested after a light (top row) and dark period (bottom row) stained with iodine.

The green biomass was measured at three time points: four, ten and fifteen weeks after planting. Five mutated lines and the RNAi line were measured relatively the parental variety: 82079 (Group 1), 104006 and 104018 (Group 2), 104010 and 104023 (Group 3) and T-2012 (RNAi) (Fig. 7a, Supplementary Fig. S8). After four weeks, the parental variety had the highest biomass, lines from Group 1 and 2 the second highest and lines from Group 3 the lowest. After ten weeks, all plants had a similar biomass, while after fifteen weeks, the lines from Group 3 had the highest biomass and had passed all other lines in growth. The parental variety plants had started to wither at time point three, which affected the biomass, most likely due to a heavy aphid infection and pest control treatment. Therefore, the values of the mutated lines as compared to the parental variety at time point three are overestimated, but still useful for comparison between the mutated lines.

Leaf tissue from the top shoots harvested at ten weeks after planting was stained with iodine after a light and dark period. As can be seen in Fig. 7b, the lines from Group 3 had no staining, showing a deficiency in transitory starch synthesis during the light period, while lines from Group 1 and 2 were similar to T-2012 and the parental variety (Fig. 7b). The lines from Group 1, 2 and 3 had the expected lack of transitory starch after the dark period. An exception was T-2012, which still stained after the dark period. It can be speculated that the granular structure of the leaf starch affects accessibility of the starch degrading enzymes, however, further studies are needed to elucidate the cause.

## Discussion

The number of alleles mutated, and the genomic structure of mutations induced in *Sbe1* and *Sbe2* of potato had a major impact on the starch quality. To develop a starch with a significant increase in chain lengths corresponding to the amylose fraction, mutations in all alleles of both genes were needed (Group 3). The lines from Group 3 were found to have starch with a reduced degree of branching from 3.0% in the control to below the detection limit of 0.1% and no amylopectin could be detected when analysed by HPAEC. Notably, the lines from Group 3 had at least one allele with an in-frame mutation in one or both of *Sbe1* and *Sbe2*. In a study by Tuncel et al.^14^, the quantity of the SBEs was reduced but not absent in lines with in-frame deletions in the *Sbe* genes. These proteins could, even with a potential loss of activity, be of importance for starch synthesis to occur. The situation has not been thoroughly investigated in potato, but several studies in cereals have shown that enzymes involved in the synthesis of starch molecules and organisation into granules are active as complexes^21–23^. The remaining fraction of SBEI and SBEII in our lines from Group 3 might be crucial to maintaining protein–protein interactions and keeping other enzymes, such as soluble starch synthases, active.

Using the applied genome-editing method, we were not able to mimic the starch qualities of high-amylose potatoes that were previously developed by transgenic gene inhibition^7,12,15^. In our study, lines with full knockout of SBEI together with two to three mutated *Sbe2* alleles (Group 2) only had a minor effect on the amylose content. None of the Group 2 lines were found to have the same increased amylose content or the very large fraction of intermediate sized chains like the starch structure found in the RNAi line T-2012 included in this study, which seem to be common among high-amylose potato starches achieved by reduced transcript levels. In a previous study, using antisense to inhibit both SBEs, a reduction of branching enzyme activity to less than 1% was found in tubers with starch containing more than 50% amylose^12^. Hence, the presence of one or two Sbe2 wild type alleles is enough for the tubers to produce starch close to wild type levels of amylopectin.The chain length distribution of debranched starches suggests that the targeted mutagenesis of *Sbe1* with or without *Sbe2* had different effects on starch structure, which has also been shown in other studies^14,24–28^. This is believed to be due to different activities of the potato SBE isoforms^25^ affecting the amylopectin structure^29^. Simultaneous suppression of SBEs in potato has been shown to induce drastic effects on starch structure^12,14,15^. Consistent with these findings, our HPSEC results for debranched starch from Groups 2 and 3 showed a loss of the short-chain amylopectin fraction and elevated fractions of amylose. Accordingly, a decreased number of short chains of DP ≤ 13 and an increased proportion of intermediate-sized chains (DP14–33) were observed for starch from Group 2 by HPAEC analysis.

Many studies on cereals have reported that suppression of SBEI alone has either no detectable effect or only a minor effect on starch structure^30^. Also previous studies in potato, antisense suppression of SBEI did not affect the amylose content or the chain length distribution of the amylopectin^30^. This is in line with our HPSEC results for Group 1 starches, which showed a similar chain length distribution of amylopectin fraction in relation to the parental variety. One exception is line 104032, which can be found similar in structure to the Group 2 starches. It can be speculated that this line might be a chimera or having one *Sbe2* allele with a very large deletion that was not amplified during the genomic analyses. Even though several attempts were made to elucidate that, only wild type alleles were found using HRFA and Sanger sequencing. When studying the starches from lines in Group 1 with higher resolution using HPAEC, an altered chain-length distribution was found, indicating that a complete knockout of SBEI alone influenced the starch structure somewhat. Moreover, light microscopy of stained starch granules from Group 1 lines revealed a minor truncation in the core of the granules.

X-ray diffraction of starch correlates with crystalline organisation of double helixes in a starch granule, while the crystallinity of granular starches is mostly attributed to the amylopectin fraction^20^. Thus, the significant loss of the amylopectin fraction in the Group 3 starches might be the reason for the considerably decreased X-ray diffraction pattern of the starches from Group 3. Moreover, the level of crystallinity has an inverse relationship to the amylose content^31^. Hence, the high-amylose feature of the potatoes from Group 3 might also contribute to the lower degree of crystallinity in the potato lines. However, the high-amylose potato starch in line T-2012, produced by RNAi, did not show a difference in the X-ray diffraction pattern compared to the parental variety.

Maltese crosses in starch granules generally indicate a higher degree of ordered structure in granules with molecules arranged in a radial pattern^32^. It is known that the apparent intensity of the birefringence is not only affected by the degree of crystallinity and granule thickness but also by the orientation of the crystallites^33^. Therefore, the complete loss of Maltese crosses in the starch from Group 3 could be attributed to a deviation in the orientation of the crystallites from its radial arrangement. The results from X-ray diffraction, polarised light microscopy and NMR spectroscopy revealed a type of starch that has the ability to organise into granules with a poorly ordered amylose crystalline arrangement, even with the considerable loss of the amylopectin fraction. These results are also in agreement with the study of amylose-only barley starch^16^.

Lines from Group 3 had a significantly decreased tuber yield, tuber size and tuber dry matter content. In a previous study of high-amylose potato lines grown in the field for several years, plants were found to be stunted in growth^15^. It was speculated that the gene inhibitions could also have an effect on diurnal starch, which might have led to a depletion in the energy turnover, or that an increase in sugars in the plant might disturb other metabolic processes. In this study, the development of the Group 3 lines was supressed during the first period after planting but those lines developed a higher green biomass than lines from Groups 1 and 2 up until senescence; despite that, the transitory starch was clearly decreased after a light period. One hypothesis could be that once tuber initiation occurs and throughout the tuber development phase, the low efficiency of channelling sugars into long-term storage as tuber starch is instead redistributed to produce green biomass.

To our knowledge, this is the first time potato starch with no detectable branching has been developed and studied. It highlights the expanded knowledge we can gain from using genome editing to study enzymatic functions and consequences of the loss of enzymatic activities on biosynthesis and plant products. This study has also raised new questions, for example, how can the self-organisation of starch into granules occur even with complete absence of branching, or are they simple molecular aggregates?

## Material and methods

### Design of targets

Genomic DNA was extracted from Desiree leaf tissue using the Gene Jet Plant Genomic DNA purification Mini Kit (Thermo Fisher Scientific, Waltham, MA) and sequenced through the Illumina TruSeq PCR-free library preparation (average fragment size 350 bp) and Illumina HiSeqX (PE 2 × 150 bp) (National Genomics Infrastructure, Stockholm, Sweden). Targets were selected in regions of exon 5 in *Sbe1* (GenBank accession no. NW_006238958.1:c2098376-2090439) and in exon 3 in *Sbe2* (GenBank accession no. NW_006238947.1: c2592132-2611729) using CRISPR RGEN Tools (http://www.rgenome.net/cas-designer) and a previously available guide design tool (http://crispr.mit.edu/). Double sgRNA targets were selected for each gene and named BE1T3 and BE1T4 as well as BE2T3 and BE2T4 (Fig. S1). All sgRNAs were designed to target allelic homologous regions, except for BE2T4 which had a mismatch at the first bp prior PAM in one of the four alleles.

### Mutagenesis and genotyping

Mutations were induced in *Solanum tuberosum* L*.* cultivar Desiree through PEG-mediated protoplast transfection of RNPs. In vitro propagation, protoplast isolation, regeneration and mutation screening and characterisation were performed as described in Andersson et al.^11^. Transfection of RNPs to isolated protoplasts was done essentially as described in Andersson et al.^35^ using 5 ug in vitro transcribed RNA preassembled with 5 ug Cas9 per target (Thermo Fisher Scientific, Waltham, MA) for lines named 82- or 0.1 nmol synthetically produced crRNA preassembled with 5 ug Cas9 per target (IDT, Coralville, IA, USA) for lines named 104-.

The transfection conditions were 40% PEG and 30 min incubation time. Care was taken to only pick one shoot from each calli. High-resolution fragment analysis (HRFA) was performed as previously described^11^ by multiplexing primers SBE1f.-HEX and SBE1_r with SBE2_3f.-FAM and stbe2exonr and screening for mutations in both genes simultaneously. Sanger sequencing (Eurofins Genomics, Ebersberg, Germany) was performed using unlabelled primers of SBE1f.-HEX and SBE1_r, SBE2_3f.-FAM and stbe2exonr as well as stbe2exonr and Stbe2exonf (Supplementary Table S1).

### Greenhouse cultivation

Thirteen genome edited lines, the parental variety Desiree and one RNAi line, T-2012 from the parental variety Dinamo developed in a previous study^7^, were grown in a greenhouse. I*n vitro* cuttings of the lines were planted in soil (Yrkesplantjord, SW Horto, Hammenhög, Sweden) in three to five biological replicates in 7.5 L pots and cultivated under controlled greenhouse conditions: 16-h day length, 18/15 °C day/night temperature, supplementary light intensity up to approximately 200 µmol s^−1^ m^−2^ photons, 60% relative humidity. The lines were grown for 5 months between 13 December and 21 May and were regularly fertilised with SW Bouyant RikaS 7-1-5 + mikro (SWHorto, Hammenhög, Sweden).

### Starch isolation, dry matter and amylose measurement

Starch was extracted from tubers harvested after senescence according to Larsson et al.^34^ with a minor modification where the time for sedimentation after extraction and each buffer washing steps were performed overnight to secure that small granules were retained. Dry matter content was measured on freshly harvested tubers as previously described^35^. Amylose content was measured on isolated starch using both an enzymatic assay and a colorimetric assay. The enzymatic assay was made using an amylose/amylopectin kit (Megazyme, Bray, Co, Ireland) according to the supplier’s instructions. The colorimetric method was based on iodine complex formation stabilized with trichloroacetic acid. The amylose content was analyzed according to Chrastil^36^ with minor modification. After solubilisation of starch with Urea-dimethylsulphoxide (UDMSO) according to Morrison and Laignelet^37^, 25 µL of sample was transferred to each of two test tubes. 5 mL 0.5% trichloroacetic acid and 50 µL 0.01 N KI-I_2_ solution were directly added for 30 min incubation at 25 °C before the absorbance measurement. A standard curve (R^2^ = 0.999) was developed using waxy potato starch (Lyckeby starch AB, Kristianstad, Sweden) and potato amylose standard (type III, Sigma Chemical Co., MO, USA) with varying amylose contents of 0%, 25%, 50%, 75% and 100%. All samples were analyzed in duplicate and results are reported as average values with standard deviation (SD) based on total starch content.

### Starch structural analysis

Starch samples solubilised in UDMSO (0.6 M urea in 90% DMSO) were used for chain-length distribution analysis by high-performance size exclusion chromatography (HPSEC) and high-performance anion exchange chromatography (HPAEC) as previously described^38–40^. The starch samples were debranched with isoamylase from *Pseudomonas sp* (EC 3.2.1.68, 500 U/mL, Megazyme, Wicklow, Ireland) and pullulanase M1 from *Klebsiella planticola* (EC 3.2.1.41, 700 U/mL, Megazyme, Wicklow, Ireland) before both analyses. The enzymes were desalted and ten-fold diluted using acetate buffer (0.01 M, pH 5.0) before applied for debranching.

For HPSEC, 300 μL each enzyme and 400 μL distilled water was mixed with 500 μL solubilised starch sample for debranching overnight at 40 °C. For HPAEC, 60 μL each enzyme and 880 μL distilled water and 400 μL sodium-acetate buffer (0.01 M, pH 5.0) was mixed with 100 μL solubilised starch sample for debranching overnight at 40 °C. For both HPSEC and HPAEC, the samples were boiled for 10 min after debranching to terminate the enzyme reaction and then filtered through a 0.45 μm nylon filter before analysis.

The HPSEC was performed as described in Andersson et al.^41^ with minor modifications. The HPSEC system has two serially connected OHpak SB-802.5 HQ columns with a guard column (Shodex, Showa Denko KK, Miniato, Japan) kept at 35 °C. The eluent was 0.1 M NaNO_3_, containing 0.02% NaN_3_ with a flow rate of 0.5 mL/min. The HPSEC is equipped with refractive index (RI) detector (Wyatt Technology Corp., Santa Barbara, CA) and multiple-angle laser light scattering detector (MALLS; Dawn DSP equipped with a He–Ne laser at 632.8 nm, Wyatt Technology Corp., Santa Barbara, CA). Data for molecular-weight determinations were analysed using ASTRA software version 4.70.07 (wyatt.com/products/software/astra.html, Wyatt Technology Corp., Santa Barbara, CA) based on a dn/dc of 0.147^41^. The angular fit was based on the Debye procedure^41^. The HPSEC columns were calibrated with Dextran T2000, Dextran T500, maltoheptaose, maltopentaose, maltotriose, and glucose (Supplementary Fig. S9). Results are given as the average of two replicates and the total area of HPSEC chromatograms were normalized between elution volume 10 mL and 17 mL, after which maltopentaose, maltotriose, and glucose were eluted (Supplementary Fig. S9).

The HPAEC (Series 4500i, Dionex Corp., Sunnyvale, CA, USA) coupled with a BioLC gradient pump and a pulsed amperometric detector (PAD) was used in this study. Separation was performed on a CarboPac PA-100 (4 × 250 mm) analytical column (Dionex, Sunnyvale USA) equipped with a guard column. The elution was performed at 25 °C with a flow rate of 1 mL/min and injection volume of 25 μL using 0.15 M NaOH (A) and 0.50 M NaOAc + 0.15 M NaOH (B) with the following gradient: 0–15 min, 15–28% eluent B; 15–45 min, 28–55% B; 45–75 min, 55–70% B; and 75–80 min 70–15% B (return to the start mixture). The column was equilibrated with 15% eluent B for 15 min between runs. The PAD response was converted to molar percentage and normalised^38^. All results are given as the average of two replicates.

### NMR spectroscopy

Starch from the parental variety and the Group 3 lines were analysed by NMR for degree of branching according to Tizzotti, et al.^42^. Starch samples (10 mg) were dissolved by heating in deuterated dimethyl sulfoxide (DMSO-*d*_6_; 600 µL) with the addition of deuterated trifluoroacetic acid (TFA-*d*_1_; 10 µL) prior to NMR analysis to avoid spectral interference with hydroxyl protons. ^1^H NMR spectra were recorded on a Bruker Avance III 600 MHz spectrometer using a 5 mm broadband observe detection SmartProbe. Spectra were acquired at 50 °C using 128 scans and a relaxation delay of 12 s and were processed with TopSpin 3.6. The degree of branching was measured as the ratio *I*_1−6_/(*I*_1−6_ + *I*_1−4_), where *I*_1−6_ is the integrated signal at 4.77 ppm and *I*_1−4_ is the integrated signal at 5.12 ppm, corresponding to H1 of glucose at the α(1 → 6) and α(1 → 4)-linkages, respectively.

### X-ray diffraction patterns

The method for X-ray diffraction analysis has been previously described^5^. Starch X-ray patterns were identified using a PANalytical X’Pert alpha1 powder X-ray diffractometer in Theta-2Theta geometry, and coupled with a focusing Johansson Ge monochromator producing pure Cu-Kα1 radiation (λ = 1.54060 Å). The starches were spread onto 1.5^2 cm^2^ Si wafers. The scanning region of the diffraction angle (2θ) was from 5° to 40°.

### Polarised light microscopic analysis of starch granules

Starch dispersions (0.5 mL, 50 mg/mL) in distilled water were freshly prepared. One drop (25 μL) of each starch dispersion was taken for microstructural analysis^5^ using a light microscope (Leica DMLB, Wetzlar, Germany) equipped with an infinity X-32 digital camera (DeltaPix, Samourn, Denmark). The images of starch granules were captured at a 20 × objective under polarised light to compare the birefringence of starch granules from lines from Group 3, 104016 (from Group 2) and the parental variety.

### Starch granule phenotyping

Starch granule distribution and phenotype was studied by staining thin tuber discs and purified starch granules with Lugol’s solution. The results were visualised with a light microscope (LeicaDMLB, Leica Microsystems, Wetzlar, Germany) and documented with an assembled camera (Leica DFC450C, Leica Microsystems, Wetzlar, Germany).

### Green biomass phenotyping

Green biomass of five of the mutated lines, 82079, 104006, 104018, 104010, 104023 as well as Desiree and T-2012, were studied in a greenhouse. The plants were grown between 7 October and 22 January under the same growth conditions as described above.

Green biomass was measured at four, ten and fifteen weeks after planting using a low-cost RGB imaging phenotyping lab published by Armoniene et al.^35^. The plants were placed on a rotating disc and images were taken as side views with a standard camera (Canon 1300D, Canon, USA) tethered to the software digiCamControl v2,1,2 (http://www.digicamcontrol.com)^43^. Four photos were taken of each plant and three biological replicates covering 360º of the plants. The images were processed using Easy Leaf Area^44^.

Top leaves were harvested at week 10, decoloured by boiling in 80% EtOH and stained with 50% Lugol’s solution.

### Sprouting

Harvested greenhouse grown potatoes were stored in the dark at 6 ℃ for five months before being incubated at 22 ℃ for 3 months. Sprout development was studied through naked eye observation once a week.

## Supplementary Information

Supplementary Information
