# Supplementary material for: Amylose starch with no detectable branching developed through DNA-free CRISPR-Cas9 mediated mutagenesis of two starch branching enzymes in potato
Source: Sci Rep. 2021 Feb 22;11:4311. doi: 10.1038/s41598-021-83462-z (PMC7900246; doi:10.1038/s41598-021-83462-z)
Supplement: Supplementary file 1 — Supplementary Information [file 41598_2021_83462_MOESM1_ESM.docx]

**Supplementary information**

**Amylose only potato starch with no detectable branching developed through DNA-free CRISPR-Cas9 mediated mutagenesis of two starch branching enzymes**

Xue Zhao^1#^, Shishanthi Jayarathna^1#^, Helle Turesson^2^, Ann-Sofie Fält^2^, Gustav Nestor^1^, Matías N. González^3,4^, Niklas Olsson^2^, Mirela Beganovic^2^, Per Hofvander^2^, Roger Andersson^1^ and Mariette Andersson^2*^

**
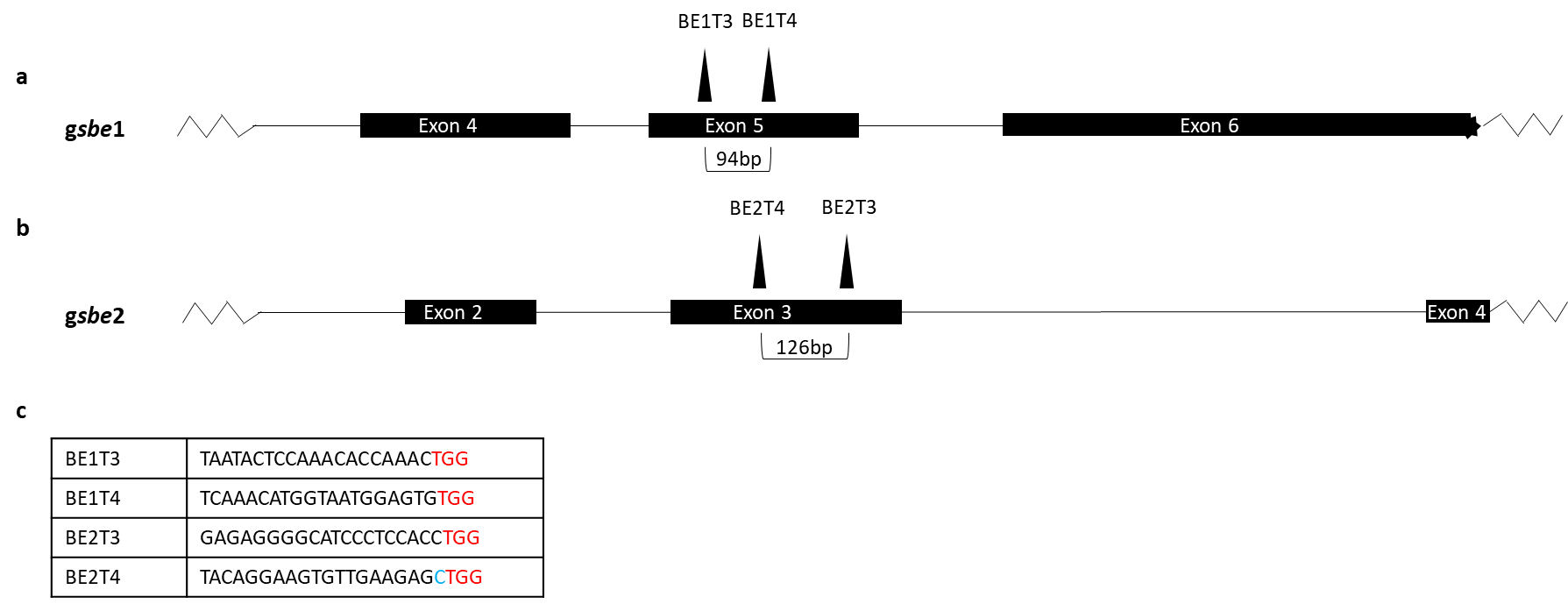
**

**Figure S1**. Schematic figure of parts of the *Sbe1* and *Sbe2* genes and the location and sequence of target regions. **a**. *gsbe1*; genomic region of *Sbe1*, target regions BE1T3 and BE1T4 and distance in bp between expected cut sites **b**. *gsbe2*; genomic region of *Sbe2*, target regions BE2T3 and BE2T4 and distance in bp between expected cut sites. **c**. Sequence of target regions, allelic variation in one of four alleles marked in blue and PAM site marked in red. Targets defined using CRISPR RGEN Tools (http://www.rgenome.net/cas-designer) and a previously available guide design tool (http://crispr.mit.edu/)

**Table S1.** Primers used for indel screening using High Resolution Fragment Analysis (HRFA) and Sanger sequencing.

| **Primer name** | **Sequence** | **Labelling (**for HRFA**)** |
| --- | --- | --- |
| SBE1f | CAATGCAGGGAAGCAGAAG | HEX |
| SBE1_3r | CTGCAAACTTTGTGGCGTCT |  |
| SBE2_3f | GGAACACGCTAGCCAGATTAAA | FAM |
| stbe2exonr | GATAGTTTGTCAAAAGGGGGTC |  |
| Stbe2exonf | TTCTTACAATTCCGAATCCCG |  |

**Table S2.** Mutation frequency of regenerated shoots**.**

| **Experiment** | **Number of regenerated shoots analysed** | **Number of shoots with at least 1 allele mutated** |
| --- | --- | --- |
| 82 | 221 | 114 |
| 104 | 68 | 49 |

**Table S3** Sequence of Group 3 lines. PAM sites are marked in red, deletions are marked with “−“ and inserts are seen in lower case letters.

| **Target** | **Line** | **Sequence** | **Indel size** |
| --- | --- | --- | --- |
| BE1 | 104010 | CCAGTT−−GTGTTTGGAGTATTAGAATTCCTGATGTTGACAGTAAGCCAGTCATTCCACACAACTCCAGAGTTAAGTTTCGTTTCAAACATGGTAAT−−−GTGTGG | (-2, -3) |
|  |  | CCAGTT−−−−−−−−−−−−−−−−−−−−−−−−−−−−−−−−−−−−−−−−−−−−−−−−−−−−−−−−−−−−−−−−−−−−−−−−−−−−−−−−−−−−−−−−−−−−−−aaaaaggcactaacggtcagattttgacctttaaaggccatctcttcttctttatttttactcaacaccattgttggacaaatttgtgttttttcctccccttttctcgattttgtctcttcagattttgaaatacccaaaagtttgacttcaacgcggcatcttatagactaaaaacttggacaGTGTGG | (-94, +186=92) |
|  |  | CCAGTT−−−TGTTTGGAGTATTAGAATTCCTGATGTTGACAGTAAGCCAGTCATTCCACACAACTCCAGAGTTAAGTTTCGTTTCAAACATGGTAATGGAacctctacctaatataaccttaagacacacttgagtaagacaagaagtcaccgcccatacaacctcttcaagcacacttaagtgttcctcaagattaccttagataaggacatttcctttacaacaGTGTGG | (-3, +126) |
|  | 104023 | CCAGTT−−−−−−−−−−−−−−−−−−−−−−−−−−−−−−−−−−−−−−−−−−−−−−−−−−−−−−−−−−−−−−−−−−−−−−−−−−−−−−−−−−−−−−−−−−−−−−cctTGTGG | (-95+3) |
|  |  | CCAGTT−−−−−−−−−−−−−−−−−−−−−−−−−−−−−−−−−−−−−−−−−−−−−−−−−−−−−−−−−−−−−−−−−−−−−−−−−−−−−−−−−−−−−−−−−−−−−−tttctgattcatgttatggatgatacctagatgtttagacgtaccttggagtgtagaaatcaattagaaaacataggtacgattcgggggaactcatatggaaagatGTGTGG | (-94, +107) |
|  |  | CCAGTT−−−−GTTTGGAGTATTAGAATTCCTGATGTTGACAGTAAGCCAGTCATTCCACACAACTCCAGAGTTAAGTTCCGTTTCAAACATGGTAATGGAaGTGTGG | (-4, +1) |
|  |  | CCAGTT−−−−−−−−−−−−−−−−−−−−−−−−−−−−−−−−−−−−−−−−−−−−−−−−−−−−−−−−−−−−−−−−−−−−−−−−−−−−−−−−−−−−−−−−−−−−−−acaaattgttaggttgtttaagatcgtccaccaattctgagattatctttcgggagtgaggtttccatgagttcactactagtcgtttcatgtctcaagatactaaaatgttgagtttctgataaactactcaaggaatccttgctcaaactatccttttaaaagaggtaactactcttgaaatatctttgaactcataaatcatttagaaatcaaagtttctcttttatcaatgtaaaaactgatacctttaggaatactccattaccatgtttgaaacgaaacttaactctggagttgtgtggaatgactggcttactgtcaacatcaggaattctaatactcGTGTGG | (-94, +345) |
| BE2 | 104010 | TACAGGAAGTGTTGA−−AGCTGGATTTTGCTTCATCACTACAACTACAAGAAGGTGGTAAACTGGAGGAGTCTAAAACATTAAATACTTCTGAAGAGACAATTATTGATGAATCTGATAGGATCAGAGAGAGGGGCATCCCTCCACCTGG | (-2) |
|  |  | TACAGGAAGTGTTGAAGAGTTGGATTTTGCTTCATCACTACAACTACAAGAAGGTGGTAAACTGGAGGAGTCTAAAACATTAAATACTTCTGAAGAGACAATTATTGATGAATCTGATAGGATCAGAGAGAGGGGCATCCCTC−ACCTGG | (-1) |
|  |  | TACAGGAAGTGTTGAAGgtcgaacagagccaaggtaagtgttctcgtgtactccttttcaagtttaagttgtggttgagctttccagctcgcatactagtacattcaatgtactgatgccagttgccctgcatcttattatgatgcagacgcaggtacccaggatcagttagcttatttaacagaagctctctgtgaaccttggattagtAGCTGGATTTTGCTTCATCACTACAACTACAAGAAGGTGGTAAACTGGAGGAGTCTAAAACATTAAATACTTCTGAAGAGACAATTATTGATGAATCTGAT-AGGATCAGAGAGAGGGGCATCCCTCCaACCTGG | (+193, +1) |
|  |  | TACAGGAAGTGTTGAAG−−CTGGATTTTGCTTCATCACTACAACTACAAGAAGGTGGTAAACTGGAGGAGTCTAAAACATTAAATACTTCTGAAGAGACAATTATTGATGAATCTGATAGGATCAGAGAGAGGGGCATCCCTCtggaagagattgaatcaaaagagaagatccttaagttcaaacttgacgatCACCTGG | (-2, +50) |
|  | 104023 | TACAGGAAGTGTTGAAGA−−−−−−−−−−−−−−−−−−−−−−−−−−−−−−−−−−−−−−−−−−−−−−−−−−−−−−−−−−−−−−−−−−−−−−−−−−−−−−−−−−−−−−−−−−−−−−−−−−−−−−−−−−−−−−−−−−−−−−−−−−−−−−−CCT | (-127) |
|  |  | TACAGGAAGTGTTGA−−AGCTGGATTTTGCTTCATCACTACAACTACAAGAAGGTGGTAAACTGGAGGAGTCTAAAACATTAAATACTTCTGAAGAGACAATTATTGATGAATCTGATAGGATCAGAGAGAGGGGCA−−−−−−−ACCTGG | (-2, -7) |
|  |  | TACAGGAAGTGTT−−−−AGCTGGATTTTGCTTCATCACTACAACTACAAGAAGGTGGTAAACTGGAGGAGTCTAAAACATTAAATACTTCTGAAGAGACAATTATTGATGAATCTGATAGGATCAGAGAGAGGGGCATCCCTC−ACCTGG | (-4, -1) |


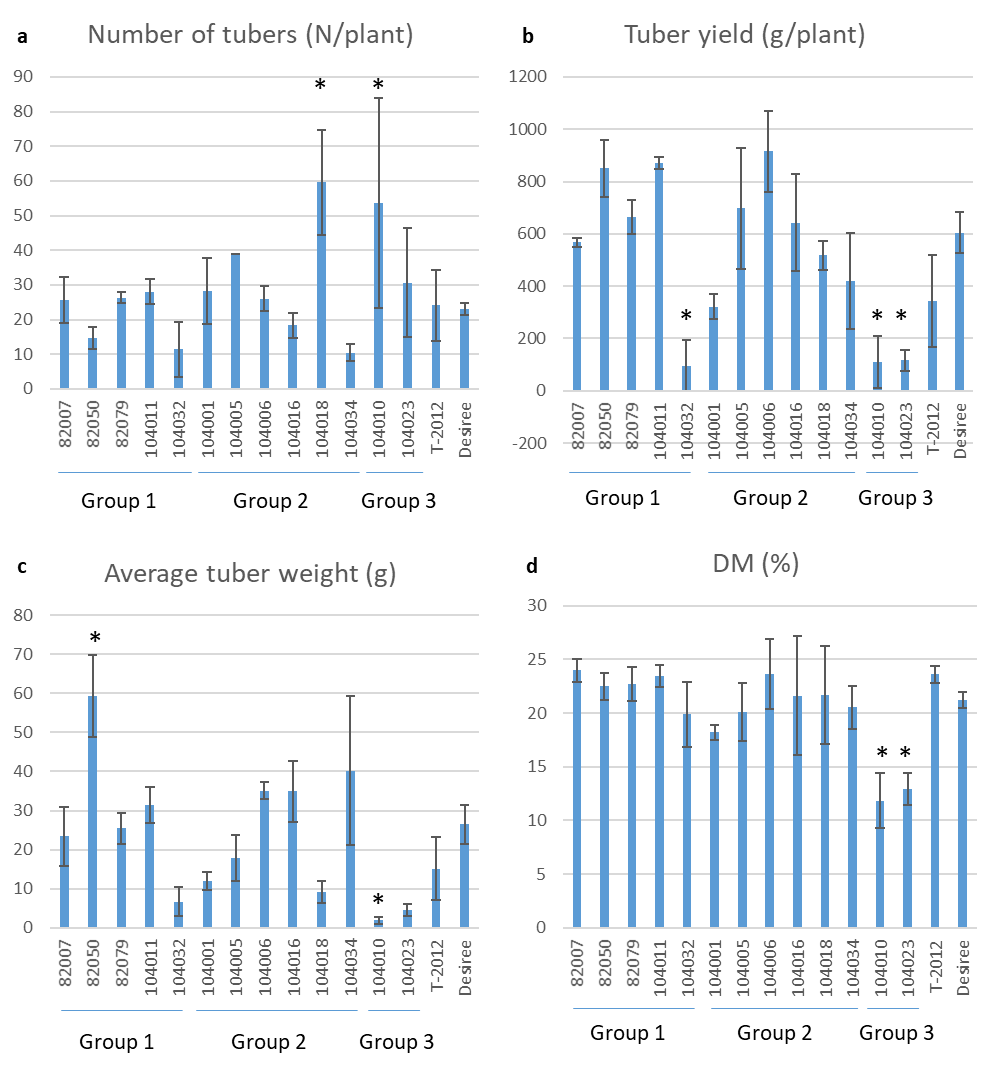


**Figure S2**. Data from Greenhouse trial. **a**. Tubers per plants **b**. Tuber yield per plant **c**. Average tuber weight **d**. Dry matter (DM). Data presented are a mean of three plants, error bar represents standard deviation (s.d). Values that differ of the parental variety Desiree by Dunnett’s test (P<0.05) are marked with *


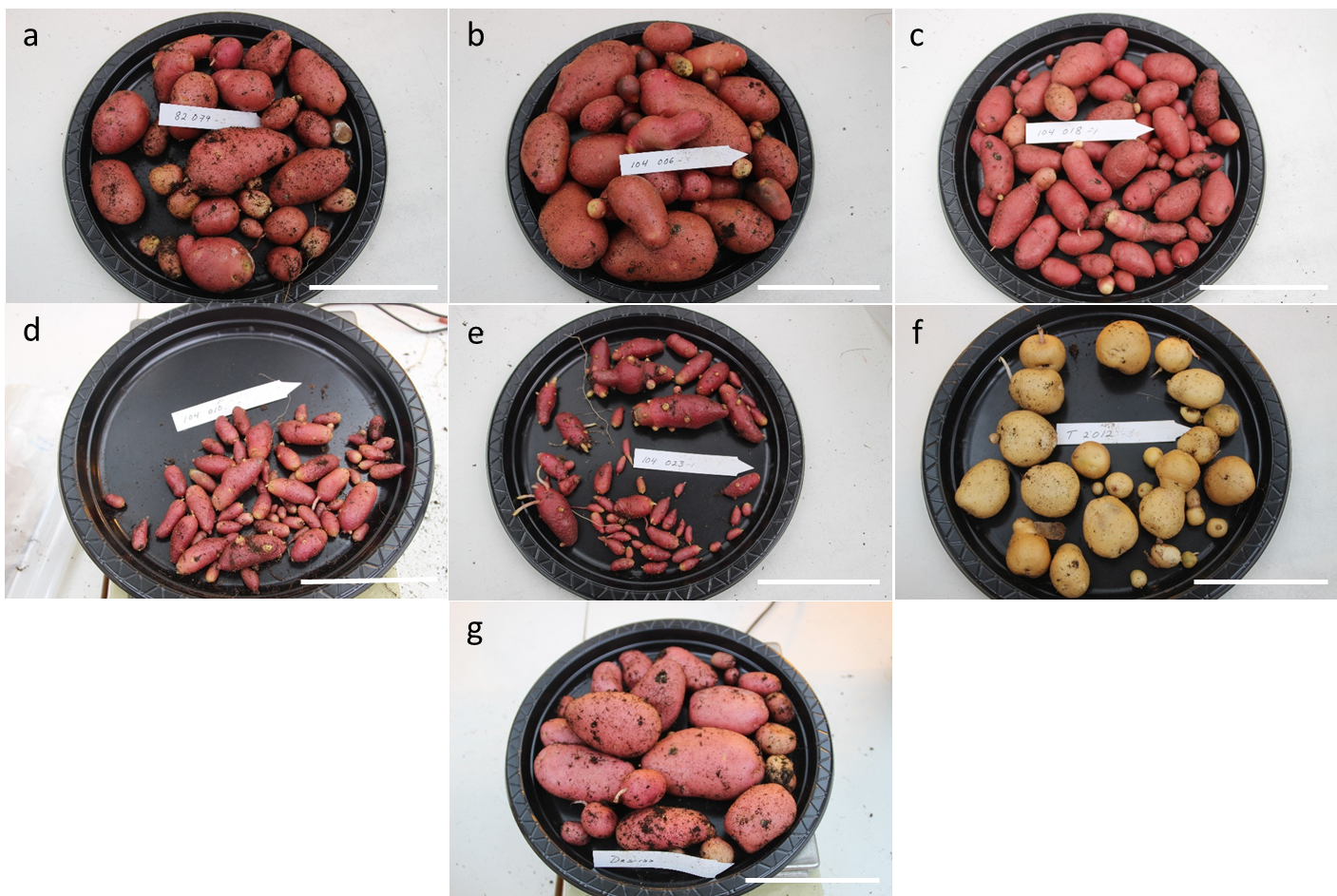


**Figure S3**. Representative harvest of tubers from one pot **a**. line 82079 (Group1) **b**. line 104006 (Group2) **c**. line 104018 (Group 2) **d**. line 104010 (Group 3) **e**. line 104023 (Group 3) **f**. line T-2012 and **g**. parental variety Desiree. Scale bar = 10 cm


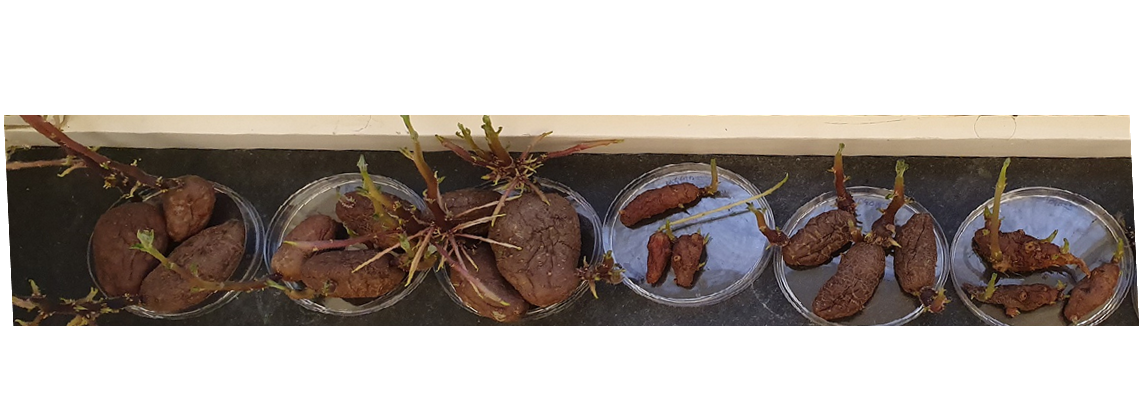
**Figure S4.** Sprouting of tuber after five months in cold storage followed by three months in room temperature. From left to right; Desiree, line 82079 (Group1), line 104006 (Group2), line 104018 (Group 2), line 104010 (Group 3) and line 104023 (Group 3).


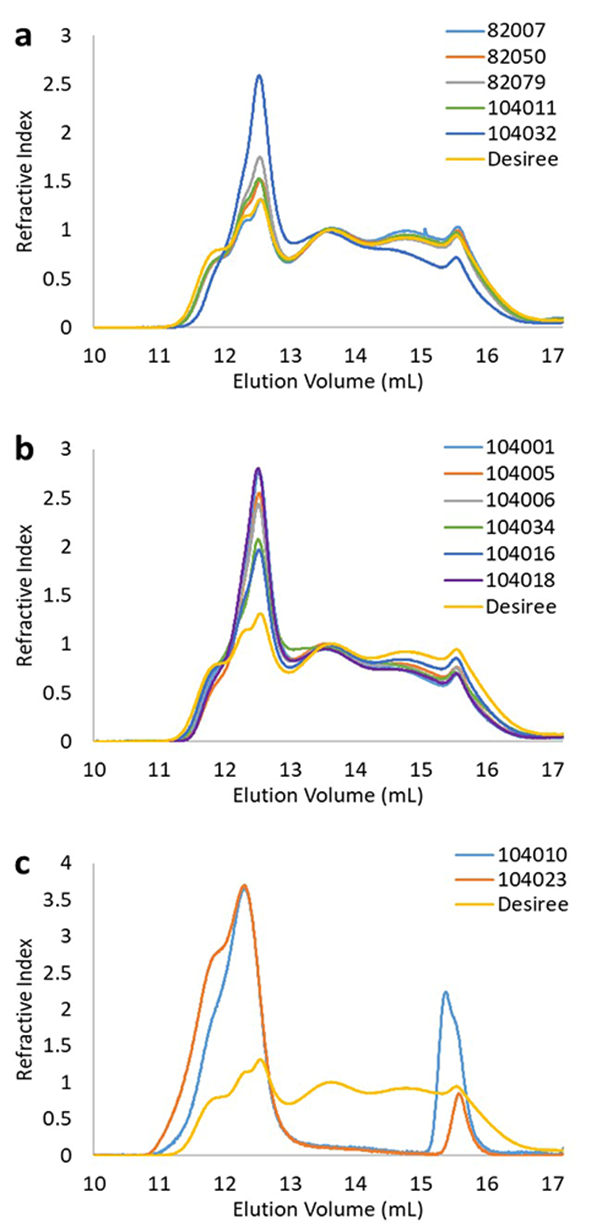


**Figure S5**. Chain length distribution of debranched starches from the potato lines. **a**. Group 1, **b.** Group 2 and **c.** Group 3 after normalisation for the peak area, analysed using HPSEC; the parental variety Desiree was included as a control starch. Software used is ASTRA software version 4.70.07 (wyatt.com/products/software/astra.html, Wyatt Technology Corp., Santa Barbara, CA).


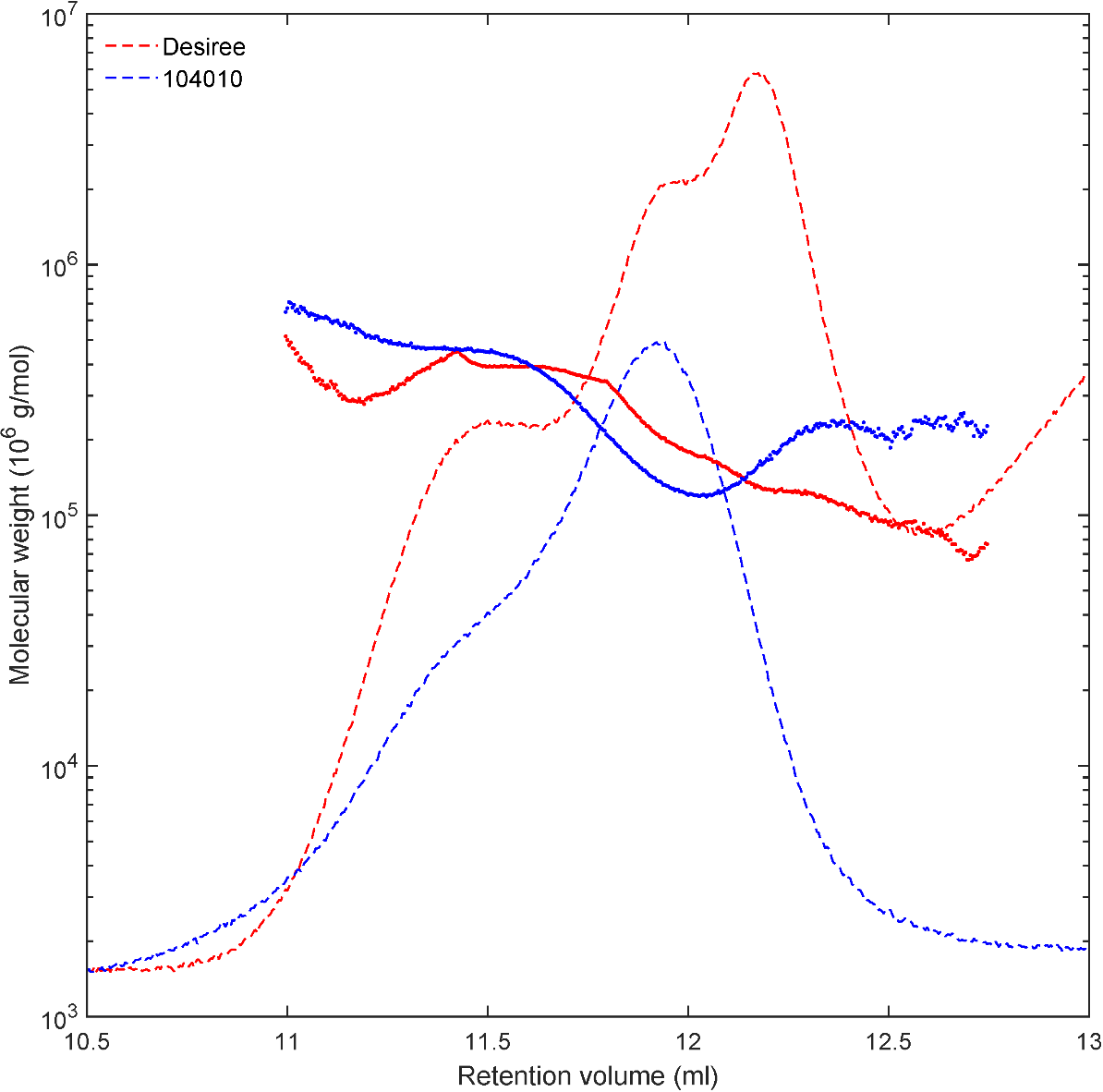


**Figure S6**. Calibration for the molecular weights based on the light scattering signal. Software used is ASTRA software version 4.70.07 (wyatt.com/products/software/astra.html, Wyatt Technology Corp., Santa Barbara, CA).


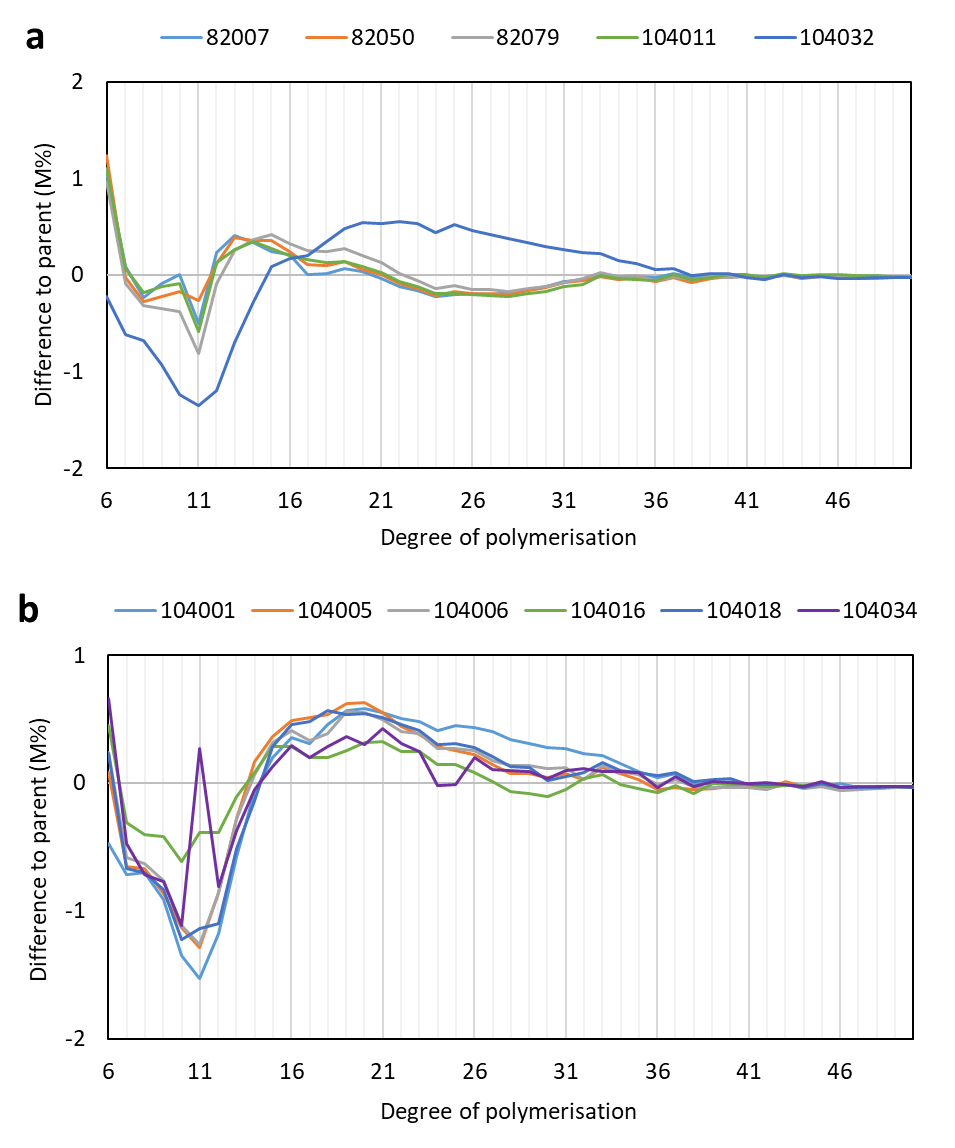


**Figure S7**. Difference plots showing changes in chain length distribution on a relative molar basis (M%) of de-branched starches from Group 1 (a) and Group 2 (b) compared to the parental potato Desiree, analysed using HPAEC.


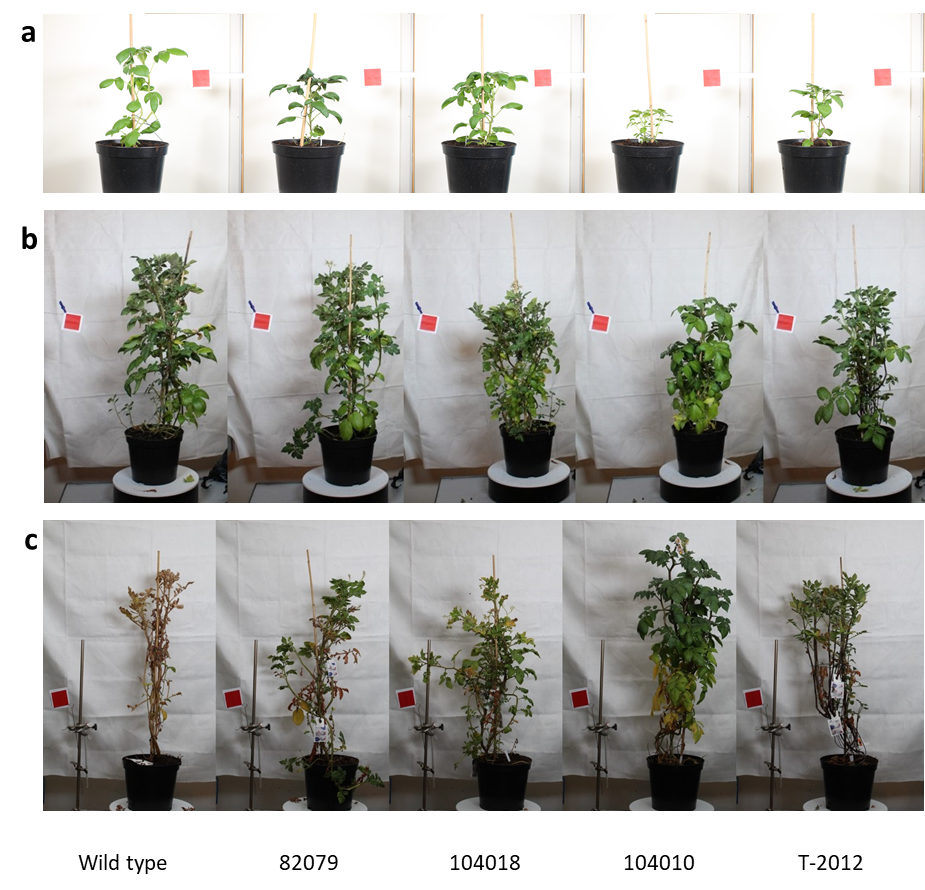


**Figure S8.** Representative photos of plants from one pot of line 82079 (Group1), line 104018 (Group 2), line 104010 (Group 3) and line T-2012 after **a.** four **b.** ten and **c.** sixteen weeks.


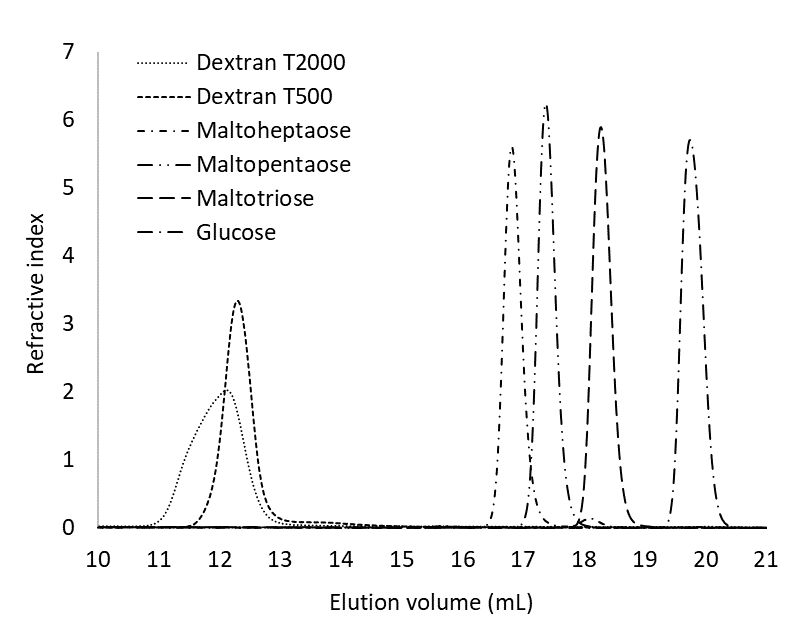


**Figure S9**. High-performance size exclusion chromatography (HPSEC) profile of standards.
